# Morphological and Morphometric Craniofacial Variations in Persian Cats: Anatomical Basis for Brachycephalic-Related Disorders

**DOI:** 10.3390/ani16132001

**Published:** 2026-06-30

**Authors:** Claudio Tagliavia, Marco Canova, Monica Prapotnich, Giulia Salamanca, Cristiano Bombardi, Angelo Peli, Annamaria Grandis

**Affiliations:** 1 Department of Veterinary Medicine, University of Teramo, 64100 Teramo, Italy; ctagliavia@unite.it; 2 Department of Veterinary Medical Sciences, University of Bologna, Ozzano dell’Emilia, 40064 Bologna, Italy; marco.canova4@unibo.it (M.C.); moniprap@gmail.com (M.P.); giulia.salamanca2@unibo.it (G.S.); cristiano.bombardi@unibo.it (C.B.); 3 Department for Life Quality Studies, University of Bologna, 47921 Rimini, Italy; angelo.peli@unibo.it

**Keywords:** brachycephaly, comparative anatomy, malocclusion, morphometry, Persian cat, skull

## Abstract

Selective breeding in Persian cats has led to extreme brachycephalic features, often associated with clinically relevant disorders. However, comprehensive anatomical studies evaluating the skull as a whole remain limited. The present study aimed to perform a morphological and morphometric comparison of the skull in Persian and Domestic Shorthair cats. Fifteen adult feline skulls, including 10 Persian and 5 Domestic Shorthair specimens, were obtained through anatomical preparation procedures and analyzed through detailed morphological observation and morphometric measurements. The results revealed consistent and marked differences between the two groups. Persian cats exhibited a rounded skull with a pronounced reduction in the facial region and frequent deviations of the craniofacial axis. Prognathism and malocclusion were observed in the majority of Persian specimens, often presenting as a reverse scissor bite. In addition, the nasal bone aperture was frequently displaced dorsally, suggesting an alteration of the anatomical relationships within the nasal region. These findings provide anatomical evidence supporting the predisposition of Persian cats to respiratory, dental, and ocular disorders and highlight the structural consequences of selective breeding on craniofacial morphology.

## Simple Summary

Persian cats are popular pets known for their round head and very short face. However, these features are the result of selective breeding and may be linked to health problems. This study examined and compared the skulls of Persian cats and more typical domestic cats to better understand these differences. The results showed that Persian cats have a much shorter facial region, a more rounded skull, and frequent misalignment of the jaws, which can lead to an abnormal bite. The position of the nasal opening was also often altered, which may affect breathing and tear drainage. These anatomical changes help explain why Persian cats are more likely to suffer from breathing difficulties, dental problems, and excessive tearing. By clearly linking skull shape to health issues, this study provides useful information for veterinarians, breeders, and pet owners, and highlights the importance of considering animal health when selecting for extreme physical traits.

## 1. Introduction

In recent decades, selective breeding has profoundly altered the morphology of several domestic species, particularly among companion animals, often favouring specific aesthetic traits at the expense of physiological balance. Among companion animals, Persian cats represent one of the most extreme examples of this process, particularly with regard to craniofacial conformation. The modern Persian cat is characterised by a markedly shortened face, a rounded skull, and a pronounced brachycephalic profile, features that have been progressively accentuated by artificial selection [1,2,3,4].

Although these morphological traits have been strongly favoured in modern breed standards, increasing attention has been directed towards their potential impact on animal health and welfare. Brachycephalic cats have been reported to exhibit an increased prevalence of several clinical conditions, including respiratory disorders, ocular disorders such as epiphora, and dental abnormalities. These conditions are commonly attributed to structural alterations affecting the upper airways, the nasolacrimal system, and the alignment of the dental arches [5,6,7,8,9,10].

Despite the growing body of anatomical studies on feline skull morphology [11,12,13,14,15], comprehensive investigations examining the Persian cat skull as an integrated morphofunctional system remain limited. Previous studies have primarily focused on specific anatomical structures or individual clinical aspects associated with brachycephaly, including the nasolacrimal duct, mandibular conformation, or encephalic structures, rather than evaluating the skull as an integrated anatomical system [16,17,18]. Consequently, a detailed anatomical assessment simultaneously integrating qualitative morphology and quantitative morphometry of the entire skull is still lacking. This integrated approach may improve our understanding of the structural relationships among different craniofacial regions and their potential contribution to the clinical conditions commonly observed in Persian cats.

A comprehensive morphological and morphometric analysis of the skull is therefore essential to better characterize the anatomical modifications associated with brachycephaly. Such an approach allows not only the identification of shape differences but also enables the assessment of proportional relationships between cranial and facial components, which are crucial for understanding their potential functional implications. In particular, the assessment of morphometric indices, including the head, cranial, facial, and palatine indices, together with craniofacial ratios, allows the evaluation of proportional relationships between neurocranial and facial regions, providing information that cannot be fully captured by isolated linear measurements alone.

The aim of the present study was to investigate the morphology and morphometry of the skull in Persian cats and to compare these findings with those observed in Domestic Shorthair cats, which represent a more physiologically typical craniofacial conformation.

## 2. Materials and Methods

The study was conducted on a total of 15 mature adult feline skulls, including 10 Persian cats (6 male and 4 female) and 5 Domestic Shorthair cats (3 male and 2 female). The exact age of the animals was not available; however, to minimize developmental variability, only mature adult specimens were included in the study, based on complete permanent dentition and full skeletal development. All specimens were obtained from animals that had died of natural causes or had been euthanised for clinical reasons unrelated to the present study. All skulls were carefully examined prior to inclusion in the study, and specimens showing evident traumatic lesions, fractures, neoplastic changes, congenital malformations, or severe pathological alterations affecting the craniofacial region were excluded. No animals were sacrificed specifically for research purposes.

The skulls were prepared using routine osteological preparation procedures. Soft tissues were manually removed, and the specimens were subjected to water maceration at a controlled temperature until complete decomposition of residual soft tissues was achieved. Following maceration, the bones were carefully cleaned, rinsed, and allowed to dry at room temperature.

### 2.1. Morphological Examination

Each specimen was then subjected to a detailed morphological examination. The analysis focused on the overall conformation of the skull, including the cranial vault, the facial skeleton, and the spatial relationships between maxillary and mandibular components. Particular attention was paid to the alignment of the craniofacial axis, the presence of asymmetries, and the morphology and orientation of the nasal and orbital regions. Facial deviations, asymmetries, and craniofacial axis alterations were qualitatively assessed through direct bilateral comparison of paired anatomical structures, including the facial skeleton, orbital margins, palatal structures, mandibular alignment, occipital condyles, and foramina, when evident. Mandibular positioning and occlusal relationships were evaluated according to standard anatomical criteria [11,12,13].

### 2.2. Craniometric Landmarks and Morphometric Measurements

In addition to the qualitative assessment, linear morphometric measurements were collected using a digital caliper with a precision of 0.01 mm.

**Figure 1:**
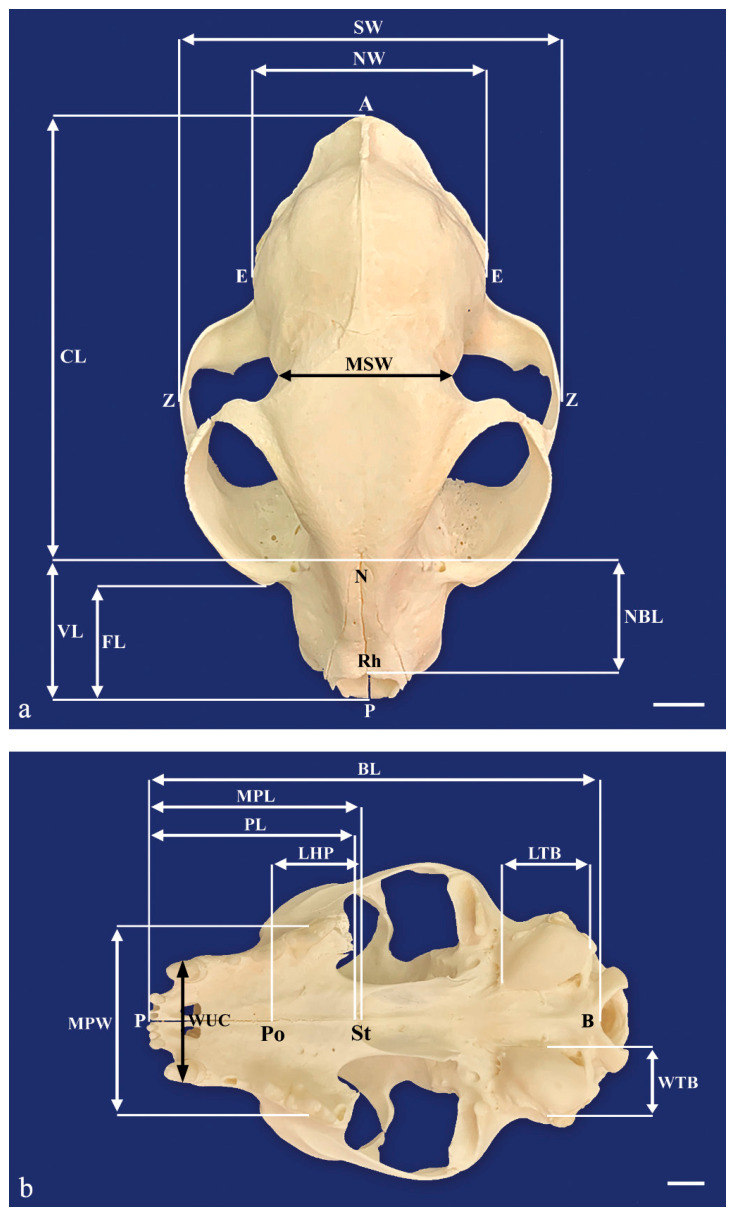
Dorsal (**a**) and ventral (**b**) views of a feline skull illustrating the craniometric landmarks and linear measurements used in the morphometric analysis. For abbreviations, see Materials and Methods. Scale bar = 1 cm.

**Figure 2:**
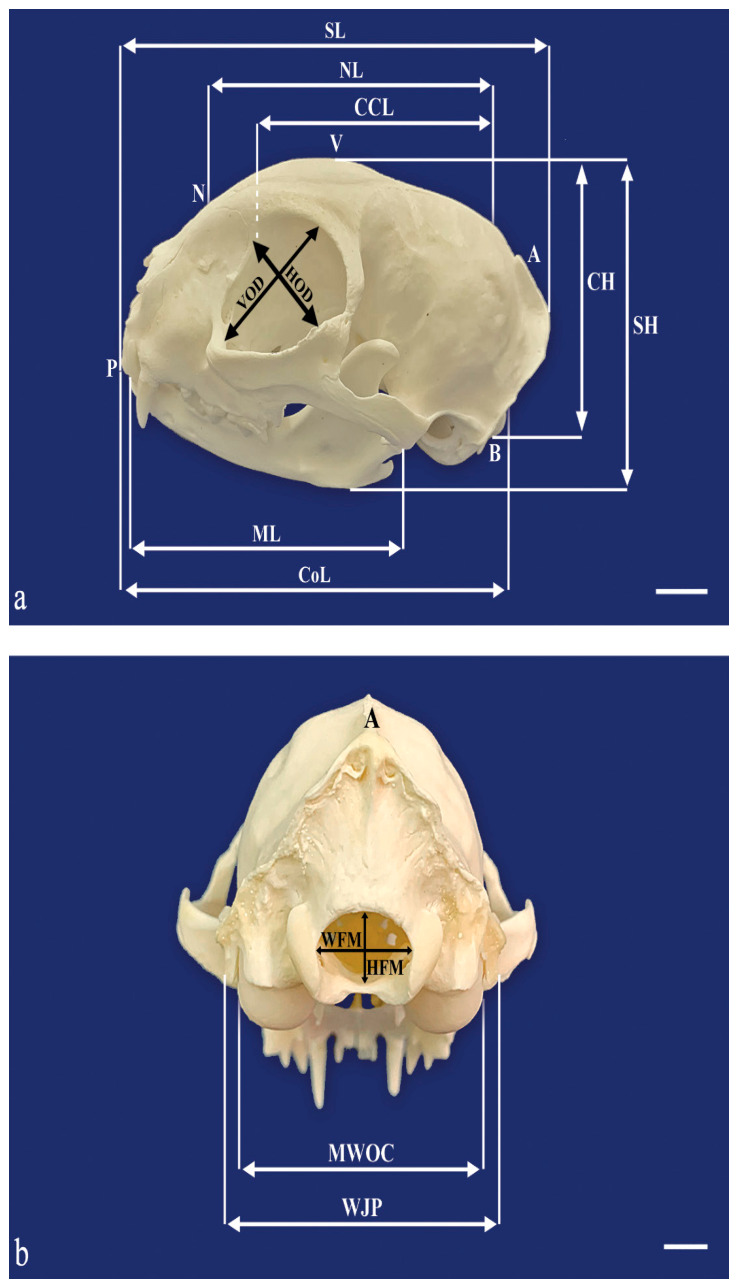
Left lateral (**a**) and caudal (**b**) views of a feline skull illustrating the craniometric landmarks and linear measurements used in the morphometric analysis. For abbreviations, see Materials and Methods. Scale bar = 1 cm.

The craniometric reference points used in the present study were based on those described by Sieslack et al. [9] and Schmidt et al. [18]. These landmarks were selected because they enable a comprehensive evaluation of the craniofacial complex, allowing assessment of proportional relationships between the neurocranium and viscerocranium and characterising anatomical changes typically associated with brachycephaly, such as facial shortening, orbital remodelling, and altered craniofacial proportions. The following craniometric landmarks were identified and used for morphometric measurements (Figure 1 and Figure 2):

- Akrokranion (A): highest point of the occipital bone
- Basion (B): midpoint of the ventral margin of the foramen magnum
- Euryon (E): the most lateral point of the cranial vault at the level of the parietal bones
- Nasion (N): median point of the frontonasal suture
- Prosthion (P): rostral point of the interincisive suture located between the roots of the upper central incisors
- Palatinoorale (Po): rostral margin of the horizontal plate of the palatine bone
- Rhinion (Rh): the most ventral point of the junction between the nasal bones
- Staphylion (St): median point of the caudal border of the hard palate
- Vertex (V): most dorsal point of the skull
- Zygion (Z): most lateral point of the zygomatic arch defining the bizygomatic diameter

Measurements were based on anatomical landmarks consistently identifiable across all specimens and selected according to established morphometric protocols for feline and carnivore skull analysis, including those described in previous studies by Onar et al. [19], Künzel et al. [14], Stacharski et al. [20], Antonelli et al. [21], Jashari et al. [22], Akbaş et al. [23], and Gundemir et al. [24]. The parameters evaluated and measurement points are reported in Figure 1 and Figure 2 and Table 1, allowing for a quantitative comparison between Persian and Domestic Shorthair cats.

All measurements were performed by a single operator to reduce inter-observer variability and were repeated twice; the mean value was used for statistical analysis in order to reduce measurement variability. The collected data were organized according to the two study groups and analyzed using statistical software (R 4.1.0, R Core Team, 2021). Data normality was assessed using the Shapiro–Wilk test prior to statistical analysis and comparisons between groups were performed using Student’s *t*-test. Statistical significance was set at *p* < 0.05. Finally, all skull specimens were photographed using a FinePix HS50 Fujifilm digital camera (Akasaka 9-chome, Minato-ku, Tokyo, Japan), and the acquired images were digitally processed using Adobe Photoshop to create the anatomical plates included in the present study. Image processing was exclusively limited to figure preparation (e.g., cropping, background adjustment, and label insertion), and no anatomical structures were digitally modified, enhanced, or altered.

## 3. Results

A total of 15 skulls were analysed in the present study, including 10 Persian and 5 Domestic Shorthair specimens. All specimens were suitable for both morphological and morphometric analysis.

No statistically significant differences in body weight were observed within each breed group. Sex distribution was recorded descriptively and sex was not included as a separate analytical variable, as no evident sex-related morphological differences were observed during the preliminary examination of the specimens and because the primary objective of the study was the comparison between breeds.

The results are summarized in Table 2, Table 3, Table 4, Table 5 and Table 6 according to the main morphofunctional patterns.

In dorsal view, Persian cats exhibited a more rounded skull shape, whereas Domestic Shorthair cats showed a more elongated conformation. In Persian cats, skull length was similar to skull width, while in Domestic Shorthair cats, skull length clearly exceeded skull width. Persian cats also showed greater minimum skull width and skull height. Accordingly, the head index was significantly higher in Persian cats (Table 2).

Several cranial measurements differed between the two groups. Persian cats showed shorter cranial, cranial cavity, neurocranial, condylobasal, and basal lengths, whereas neurocranial width was greater. In contrast, cranial height did not differ significantly between groups. Accordingly, cranial and basal indices were significantly higher in Persian cats (Table 2).

In the facial region, Persian cats exhibited shorter viscerocranial and facial lengths, as well as shorter nasal bones. Facial deviation was observed in 5 out of 10 Persian cats, and a depression at the frontonasal junction was observed in 8 out of 10 specimens. The facial index was higher in Persian cats, while craniofacial ratios did not differ significantly between groups (Table 3).

Palatal measurements also differed between groups. Persian cats exhibited shorter palatal dimensions, whereas maximum palatal width did not differ significantly between groups. Consequently, palatine indices were higher in Persian cats and palatal asymmetry was observed in 5 out of 10 specimens (Table 4).

In the mandibular region, prognathism was observed in 9 out of 10 Persian cats (90%) and in 1 out of 5 Domestic Shorthair cats (20%). Reverse scissor bite and open bite were observed in Persian cats, while mandibular length was shorter and width at the coronoid processes was greater compared with Domestic Shorthair cats (Table 4).

Dental abnormalities were observed in both groups and were more prevalent and heterogeneous in Persian cats. In particular, prognathism was observed in 9/10 Persian cats (90%), while open bite was observed in 4/10 specimens (40%). Persian cats also exhibited reverse scissor bite and altered tooth alignment. Erosions and structural remodeling mainly involved the canines, carnassial teeth, molars, and their corresponding alveoli. Reduced width at the upper canines and variations in tooth alignment were recorded (Table 4).

In the orbital region, Persian cats showed a more rounded orbital opening and a greater horizontal orbital diameter, whereas vertical orbital diameter did not differ significantly between groups. Orbital asymmetry was observed in 3 out of 10 Persian cats and in 1 out of 5 Domestic Shorthair cats. A complete orbital margin was observed in 8 out of 10 Persian cats and in 2 out of 5 Domestic Shorthair cats (Table 5).

In the caudal region of the skull, most measurements (length of the tympanic bulla, width of the tympanic bulla and width of the jugular processes) did not differ significantly between groups, indicating substantial preservation of these anatomical structures (Table 6).

Regarding foramina and openings, most measurements did not differ significantly between groups. Persian cats exhibited a greater height of the foramen magnum, whereas its width did not differ significantly. In most Persian cats, the ventral margin of the nasal aperture was positioned at or above the level of the ventral orbital margin, whereas in Domestic Shorthair cats it was positioned more ventrally. Asymmetries of foramina were observed in some specimens (Table 6).

## 4. Discussion

**Figure 3:**
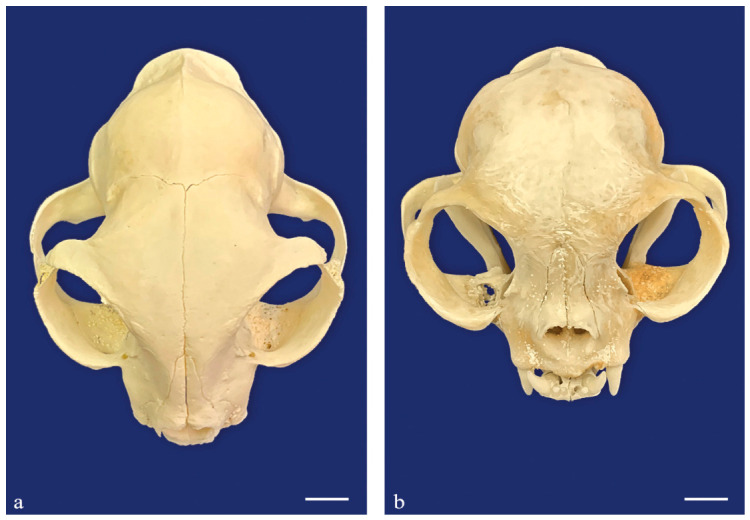
Dorsal view of the skull. (**a**) Domestic Shorthair cat; (**b**) Persian cat. Note the more elongated skull shape in the Domestic Shorthair cat and the rounded craniofacial conformation in the Persian cat. Scale bar = 1 cm.

**Figure 4:**
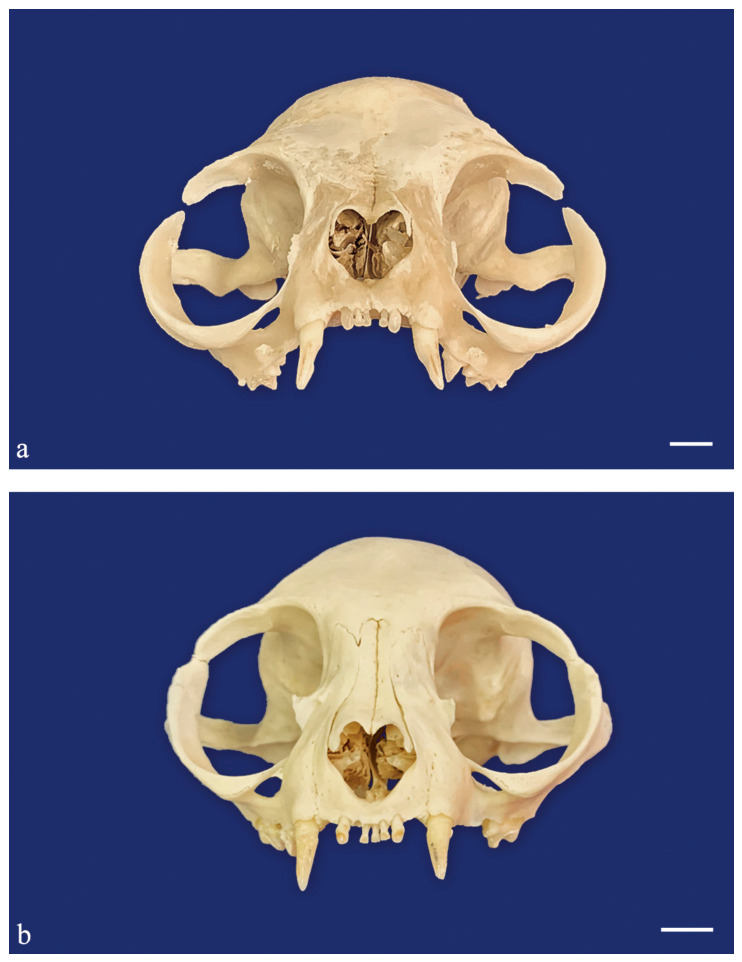
Rostral view of the skull of Persian cats. (**a**,**b**) Representative specimens showing dorsal displacement of the nasal aperture, shortening of the facial skeleton and a rounded orbital opening. Scale bar = 1 cm.

**Figure 5:**
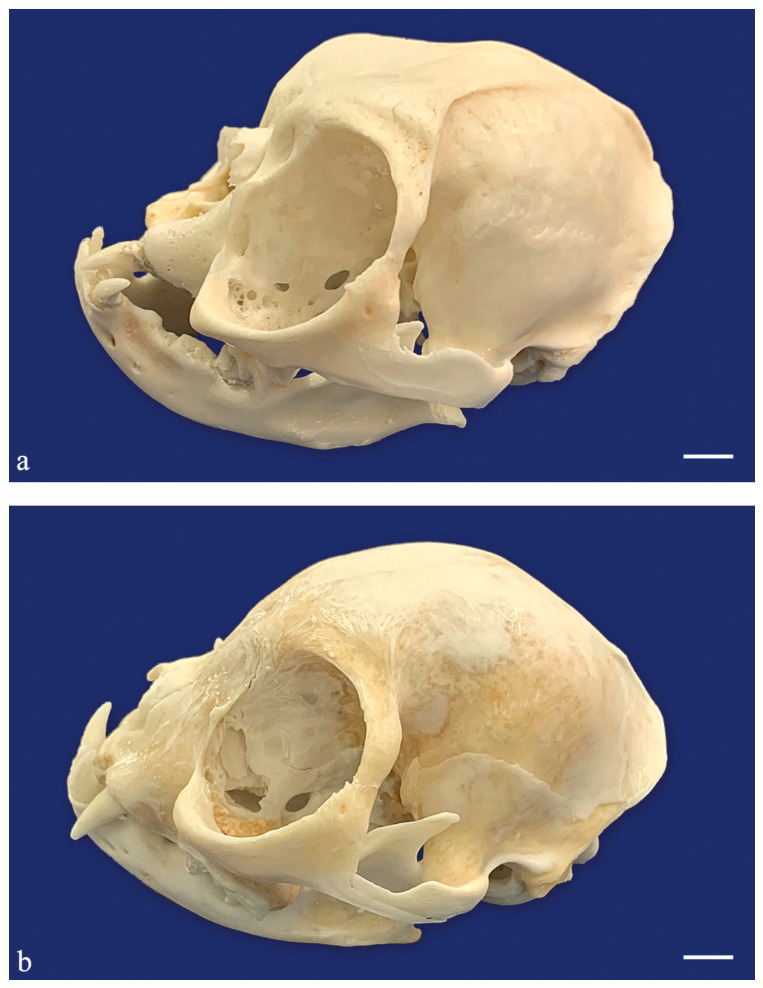
Left lateral view of the skull of Persian cats. (**a**,**b**) Representative specimens showing mandibular prognathism and dental malocclusion associated with brachycephalic craniofacial conformation. Scale bar = 1 cm.

**Figure 6:**
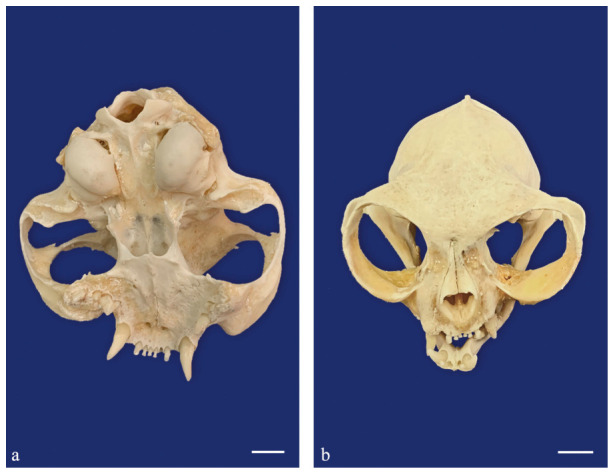
Skull of a Persian cat. (**a**) Ventral view showing palatal asymmetry, maxillary rotation, and abnormal dental development. (**b**) Rostral view showing disproportionate development of the maxillary and mandibular components associated with reverse scissor bite. Scale bar = 1 cm.

**Figure 7:**
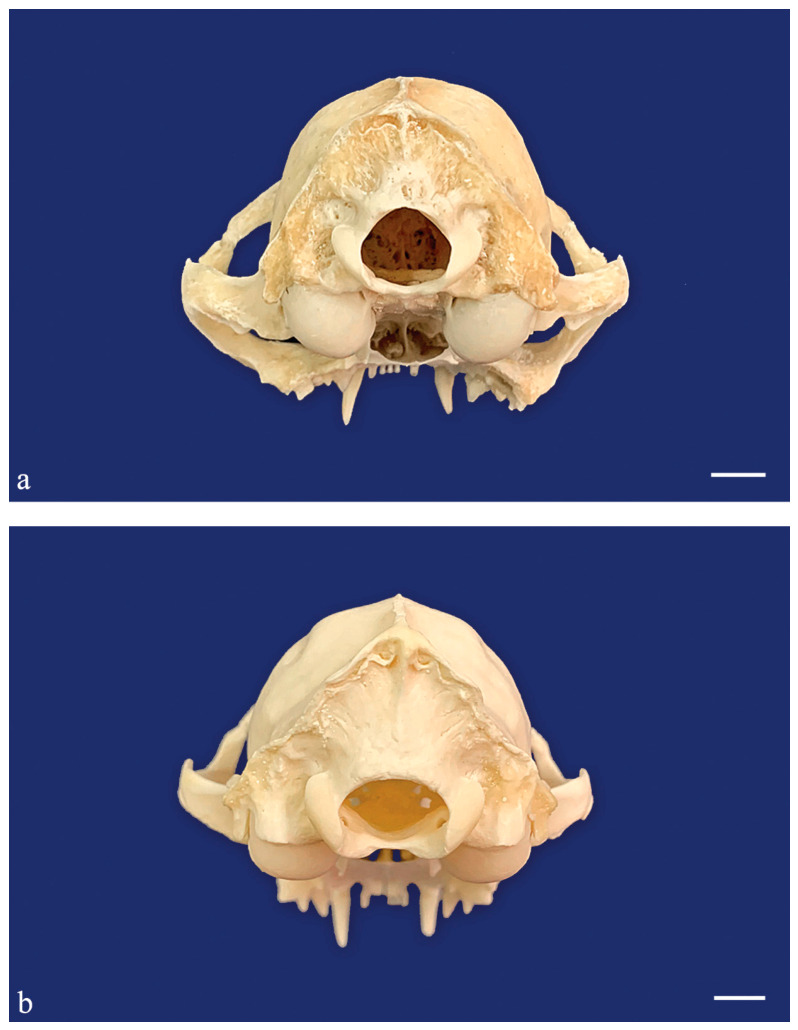
Caudal view of the skull. (**a**) Persian cat; (**b**) Domestic Shorthair cat. Note the more rounded shape of the foramen magnum in the Persian cat. Scale bar = 1 cm.

The findings of this study demonstrate that Persian cats exhibit marked craniofacial modifications compared with Domestic Shorthair cats, particularly in terms of skull shape, facial length, and cranial proportions (Figure 3, Figure 4, Figure 5, Figure 6 and Figure 7). In addition to confirming previously described brachycephalic characteristics, this study provides a comprehensive morphofunctional assessment integrating qualitative anatomical observations, linear morphometric measurements and proportional indices. Several craniofacial asymmetries and dental alterations were systematically documented, further expanding current knowledge of the anatomical variability associated with brachycephaly in Persian cats. In dorsal view, Persian cats appeared to have a more rounded skull shape (Figure 3), whereas Domestic Shorthair cats exhibited a more elongated conformation, consistent with previous observations reported by Schmidt et al. [18] and Stacharski et al. [20]. The rounded skull conformation observed in Persian cats is consistent with previous morphometric studies, which identify this breed as one of the most brachycephalic among domestic felines [9,20].

The reduction in skull length combined with increased cranial width reflects a general modification of craniofacial proportions rather than a localized alteration (Figure 3). Similar patterns have been described in previous studies, where brachycephalic skulls are characterized by shortening along the rostrocaudal axis and expansion in the transverse plane [9,20]. The significantly higher cranial indices observed in Persian cats further support this morphological configuration.

The shortening of the facial skeleton represents one of the key findings of this study. Reduced viscerocranial length and nasal bone length confirm that brachycephaly primarily affects the facial region (Figure 3). The disproportionate reduction in facial dimensions relative to overall skull dimensions, also reflected in the morphometric indices, further emphasises that brachycephaly in Persian cats primarily results from compression of the facial skeleton rather than a uniform reduction in the entire skull. The degree of facial shortening observed in this study is consistent with previous morphometric investigations, which similarly identified a marked reduction in the facial skeleton as a defining feature of the Persian breed [9,18]. The relatively high variability observed for facial length in Persian cats likely reflects the heterogeneous expression of brachycephalic traits currently present within the breed. Previous investigations have shown that facial shortening is associated with deformation of adjacent cranial structures and may reflect coordinated alterations in craniofacial development [14,18].

These structural modifications are likely to have direct and clinically relevant functional implications. The dorsal displacement of the nasal aperture observed in Persian cats (Figure 4) is consistent with previous descriptions of altered upper airway anatomy in brachycephalic species [8,16]. Such changes have been associated with increased airway resistance and respiratory disorders, including brachycephalic airway syndrome [8,9].

The reduction in palatal length, combined with the maintenance of palatal width, results in a modified palatal configuration. This finding supports previous studies showing that brachycephalic skull morphology leads to reduced maxillary space and contributes to dental crowding and malalignment [23,24]. The significantly higher palatine indices observed in Persian cats are consistent with this structural reorganization.

Mandibular and dental abnormalities were particularly evident in Persian cats (Figure 5 and Figure 6). The high prevalence of prognathism and malocclusion observed in this study (Figure 5), often presenting as reverse scissor bite (Figure 6), is in agreement with previous studies describing occlusal disorders in brachycephalic breeds [5,8]. Although the prevalence observed in the present sample was particularly high (90%), direct comparisons with previous studies are difficult because quantitative prevalence data are seldom reported. Nevertheless, previous studies consistently identify prognathism, malocclusion, and dental misalignment as common features of Persian cats, supporting the view that these abnormalities are integral components of the brachycephalic phenotype. These alterations are likely related to disproportionate development between maxillary and mandibular components, as previously suggested in the literature.

Orbital morphology also differed between groups, with Persian cats showing a more rounded orbital opening and increased horizontal diameter (Figure 1 and Figure 4). These findings are consistent with studies reporting greater ocular exposure in brachycephalic animals, which has been associated with reduced corneal sensitivity and increased susceptibility to corneal lesions [5,9]. Such anatomical characteristics may contribute to the high prevalence of ophthalmological disorders reported in Persian cats.

In contrast, the caudal region of the skull showed limited variation between the two groups (Figure 7). Most measurements in this region did not differ significantly, suggesting that the effects of selective breeding are more pronounced in the rostral and facial regions. However, Persian cats exhibited a more rounded foramen magnum (Figure 7). Similar observations have been reported in previous morphometric analyses of feline skulls [20].

The analysis of foramina and openings revealed only limited differences between the two groups. Nevertheless, the altered position of the nasal aperture in Persian cats may influence the course of the nasolacrimal duct (Figure 4). Previous studies have demonstrated that brachycephalic cats often present anatomical variations in the nasolacrimal system, including shortened or displaced ducts, which may impair tear drainage and contribute to epiphora [16].

Overall, these findings demonstrate that brachycephaly in Persian cats is not limited to isolated craniofacial modifications but involves a global reorganisation of skull morphology (Figure 3, Figure 4, Figure 5, Figure 6 and Figure 7). Such structural changes provide a clear anatomical basis for the clinical conditions frequently reported in this breed, including respiratory, ocular, and dental disorders [5,8,9]. These findings highlight the importance of integrating anatomical knowledge into breeding selection, clinical evaluation, and welfare-oriented approaches in feline medicine.

These results highlight the need for increased awareness of the anatomical consequences of selective breeding and support the importance of integrating morphological data into breeding strategies aimed at improving animal welfare. Some limitations should be acknowledged. The relatively small sample size, particularly for the Domestic Shorthair group, may have influenced the statistical significance of some comparisons. Moreover, given the relatively high number of morphometric variables analyzed, findings with marginal statistical significance should be interpreted with caution, as multiple comparisons may increase the risk of type I errors. In addition, the use of skeletal specimens did not allow evaluation of soft tissues and functional aspects. Furthermore, the study relied on conventional linear morphometric measurements and did not incorporate three-dimensional imaging or geometric morphometric approaches, which could provide a more comprehensive assessment of craniofacial shape variation and spatial relationships among anatomical structures. Future studies integrating imaging techniques and clinical data would be useful to further investigate the relationship between skull morphology and functional outcomes in brachycephalic cats.

## 5. Conclusions

In conclusion, the present study demonstrates that Persian cats exhibit marked craniofacial modifications involving both the neurocranial and facial regions. The morphometric and morphological differences identified compared with Domestic Shorthair cats provide further evidence that brachycephaly in this breed is associated with a global reorganization of skull architecture rather than isolated anatomical changes. Several of the observed features, including shortening of the facial skeleton, altered orbital morphology, mandibular prognathism, and dorsal displacement of the nasal aperture, may contribute to the clinical disorders frequently reported in brachycephalic cats.

## Figures and Tables

**Table 1:** Craniometric landmarks, morphometric parameters, and anatomical reference points used for skull measurements in Persian and Domestic Shorthair cats. The corresponding craniometric landmarks and anatomical views are illustrated in Figure 1 and Figure 2.

Measurement and Index Points	
**Skull length (SL)**	From Akrokranion to Prosthion
**Skull width (SW)**	From Zygion to Zygion
**Minimum skull width (MSW)**	Width at the postorbital narrowing
**Skull height (SH)**	From the lower corner of the mandible to the vertex
**Head index**	Maximum zygomatic width/head length × 100
**Cranial length (CL)**	From Akrokranion to Nasion
**Cranial cavity length (CCL)**	From Basion to the cribriform plate
**Cranial height (CH)**	From Basion to the Vertex
**Neurocranial length (NL)**	From Basion to Nasion
**Neurocranial width (NW)**	From Euryon to Euryon
**Condylobasal length (CoL)**	From Prosthion to the caudal margin of the occipital condyles
**Basal length (BL)**	From Basion to Prosthion
**Cranial index**	Maximum width of the neurocranium/length of the skull × 100
**Basal index**	Maximum width of the neurocranium/basal length × 100
**Viscerocranial length (VL)**	From Prosthion to Nasion
**Facial length (FL)**	From Prosthion at the oral margin of the orbits (median)
**Nasal bone length (NBL)**	From nasion to Rhinion
**Facial index**	Maximum zygomatic width/viscero-cranial length × 100
**Craniofacial ratios**	Cranial length/viscero-cranial length × 100
**Palatal length (PL)**	From Prosthion to the midpoint of the line connecting the deepest points of the choanae
**Median palatal length (MPL)**	From Prosthion to Staphylion
**Length of the horizontal part of the palatine bone (LHP)**	From Staphylion to Palatinoorale
**Maximum palatal width (MPW)**	Maximum width measured at the distal ends of the alveoli of the fourth premolars
**Palatine index**	Maximum width of the palate/median length of the palate × 100
**Mandibular length (ML)**	From the condylar process to the interdental space between the central incisors
**Width at the coronoid processes (WCP)**	Transverse distance measured between the coronoid processes
**Width at the upper canines (WUC)**	Transverse distance measured between the outermost points of the upper canines
**Horizontal orbital diameter (HOD)**	Horizontal diameter measured between the medial and lateral margins of the orbital opening
**Vertical orbital diameter (VOD)**	Vertical diameter measured between the dorsal and ventral margins of the orbital opening
**Length of the tympanic bulla (LTB)**	Rostrocaudal distance measured between the most rostral and caudal points of the tympanic bulla
**Width of the tympanic bulla (WTB)**	Laterolateral distance measured across the tympanic bulla
**Width of the jugular processes (WJP)**	Transverse distance measured between the lateral margins of the right and left jugular processes
**Maximum width of the occipital condyles (MWOC)**	Maximum transverse distance measured between the outermost lateral points of the occipital condyles
**Height of the foramen magnum (HFM)**	Dorsoventral distance measured between the dorsal and ventral margins of the foramen magnum
**Width of the foramen magnum (WFM)**	Laterolateral distance measured between the lateral margins of the foramen magnum

**Table 2:** Morphometric comparison of overall skull conformation and cranial proportions between Persian and Domestic Shorthair cats.

Anatomical Region/Parameter	Domestic Shorthair Cats	Persian Cats	*p*-Value	Morphofunctional Pattern
**Overall skull conformation in dorsal view**	More elongated skull shape	More rounded skull shape	-	**Skull shape and cranial proportions**
**Skull length**	98.55 ± 6.13 mm	75.43 ± 5.40 mm	4.57 × 10^−6^	
**Skull width**	69.01 ± 2.54 mm	74.71 ± 2.76 mm	0.001	
**Relationship between skull length and skull width**	Skull length exceeded	Skull length and skull width were similar	-	
**Minimum skull width**	31.72 ± 2.44 mm	39.76 ± 2.25 mm	2.50 × 10^−5^	
**Skull height**	49.34 ± 2.45 mm	53.32 ± 2.21 mm	0.01	
**Head index**	70.18 ± 3.87	99.43 ± 7.10	1.14 × 10^−6^	
**Cranial length**	75.18 ± 5.97 mm	57.77 ± 2.80 mm	2.76 × 10^−6^	
**Cranial cavity length**	54.85 ± 3.66 mm	40.14 ± 3.86 mm	8.47 × 10^−6^	
**Cranial height**	39.23 ± 3.16 mm	42.42 ± 3.13 mm	0.09	
**Neurocranial length**	71.18 ± 3.81 mm	54.64 ± 3.03 mm	4.79 × 10^−7^	
**Neurocranial width**	42.68 ± 1.24 mm	48.34 ± 1.93 mm	5.09 × 10^−5^	
**Condylobasal length**	90.46 ± 5.21 mm	68.88 ± 4.04 mm	6.94 × 10^−7^	
**Basal length**	51.39 ± 2.49 mm	77.23 ± 7.61 mm	4.24 × 10^−6^	
**Cranial index**	57.04 ± 4.47	83.86 ± 5.62	4.41 × 10^−7^	
**Basal index**	51.39 ± 2.49	77.23 ± 7.61	6.14 × 10^−6^	

**Table 3:** Morphometric comparison of the facial region and craniofacial relationships between Persian and Domestic Shorthair cats.

Anatomical Region/Parameter	Domestic Shorthair Cats	Persian Cats	*p*-Value	Morphofunctional Pattern
**Viscerocranial length**	36.61 ± 2.39 mm	26.25 ± 4.90 mm	0.001	**Shortening of the facial skeleton and altered upper airway anatomy**
**Facial length**	11.55 ± 1.74 mm	2.46 ± 5.77 mm	0.005	
**Facial deviation**	Not reported	Observed in 5/10 specimens (50%)	-	
**Nasal bone length**	21.46 ± 1.60 mm	11.56 ± 4.90 mm	0.001	
**Depression at the frontonasal junction**	Not reported	Observed in 8/10 specimens (80%)	-	
**Facial index**	189.10 ± 13.37	294.70 ± 63.36	0.003	
**Craniofacial ratio**	205.50 ± 14.12	226.50 ± 40.54	0.29	
**Position of the ventral margin of the nasal aperture**	Positioned more ventrally than the ventral orbital margin	Positioned at or above the level of the ventral orbital margin in most specimens	-	

**Table 4:** Morphometric comparison of palatal, mandibular, and dental parameters between Persian and Domestic Shorthair cats.

Anatomical Region/Parameter	Domestic Shorthair Cats	Persian Cats	*p*-Value	Morphofunctional Pattern
**Palatal length**	37.54 ± 2.19 mm	28.75 ± 2.02 mm	3.20 × 10^−6^	**Modified palatal conformation and mandibular/dental abnormalities**
**Median palatal length**	38.31 ± 2.48 mm	29.84 ± 2.29 mm	1.78 × 10^−5^	
**Measurements of the horizontal part of the palatine bone**	15.88 ± 1.70 mm	12.96 ± 1.53 mm	0.005	
**Maximum palatal width**	40.48 ± 1.81 mm	41.69 ± 1.73 mm	0.23	
**Palatine index**	105.85 ± 5.20	140.10 ± 7.04	2.93 × 10^−7^	
**Palatal asymmetry**	Not reported	Observed in 5/10 specimens	-	
**Prognathism**	Observed in 1/5 specimens (20%)	Observed in 9/10 specimens (90%)	-	
**Malocclusion**	Not reported	Reverse scissor bite observed in 9/10 specimens (90%) and open bite in 4/10 specimens (40%)	-	
**Mandibular length**	62.72 ± 3.48 mm	54.04 ± 3.99 mm	0.001	
**Width at the coronoid processes**	51.20 ± 3.85 mm	61.93 ± 3.06 mm	5.21 × 10^−5^	
**Dental abnormalities**	Missing alveoli: 2/5 (40%); Erosions observed in 2/5 specimens (40%)	Missing alveoli observed in 6/10 specimens (60%); Erosions observed in 4/10 specimens (40%); structural thickening in 3/10 specimens (30%)	-	
**Width at the upper canines**	23.09 ± 1.14 mm	20.58 ± 1.92 mm	0.02	
**Variations in tooth alignment**	Observed	Observed	-	

**Table 5:** Morphometric comparison of orbital morphology between Persian and Domestic Shorthair cats.

Anatomical Region/Parameter	Domestic Shorthair Cats	Persian Cats	*p*-Value	Morphofunctional Pattern
**Orbital opening**	Less rounded than in Persian cats	More rounded orbital opening	-	**Orbital morphology and ophthalmological disorders**
**Horizontal orbital diameter**	24.37 ± 0.79 mm	25.66 ± 0.73 mm	0.01	
**Vertical orbital diameter**	28.48 ± 1.74 mm	28.61 ± 1.34 mm	0.89	
**Orbital asymmetry**	Observed in 1 out of 5 (20%)	Observed in 3 out of 10 (30%)	-	
**Complete orbital margin**	Observed in 2 out of 5 (40%)	Observed in 8 out of 10 (80%)	-	

**Table 6:** Morphometric comparison of the caudal skull region between Persian and Domestic Shorthair cats.

Anatomical Region/Parameter	Domestic Shorthair Cats	Persian Cats	*p*-Value	Morphofunctional Pattern
**Caudal region of the skull**	Most measurements did not differ significantly between groups	Most measurements did not differ significantly between groups	-	**Additional findings not directly included in the main morpho-functional patterns**
**Length of the tympanic bulla**	18.74 ± 1.09 mm	17.63 ± 1.55 mm	0.18	
**Width of the tympanic bulla**	10.70 ± 1.38 mm	9.95 ± 1.09 mm	0.27	
**Width of the jugular processes**	41.07 ± 2.07 mm	41.54 ± 2.25 mm	0.70	
**Maximum width of the occipital condyles**	22.11 ± 1.82 mm	20.88 ± 0.95 mm	0.047	
**Height of the foramen magnum**	11.38 ± 0.63 mm	13.85 ± 1.32 mm	0.002	
**Width of the foramen magnum**	13.22 ± 0.54 mm	13.58 ± 0.68 mm	0.33	
**Asymmetries of foramina**	Observed in some specimens	Observed in some specimens	-	

## Data Availability

The data presented in this study are available on request from the corresponding author.
